# Supplementary material for: Detrimental effects of heat stress on grain weight and quality in rice (Oryza sativa L.) are aggravated by decreased relative humidity
Source: PeerJ. 2021 Apr 9;9:e11218. doi: 10.7717/peerj.11218 (PMC8040870; doi:10.7717/peerj.11218)
Supplement: Supplemental Information 1 [file peerj-09-11218-s001.docx]

Table S1 Heading date, average daily temperature during 15 days after heading(T15), average daily relative humidity during 15 days after heading (RH15), grain weight, head rice rate and chalkiness of each cultivar in field trial in 2016.

| Cultivars | Phases | Heading date (d-m) | T15 (°C) | RH15 (%) | Grain weight (g pre 1000 grains) | Head rice rate (%) | Chalkiness degree (%) |
| --- | --- | --- | --- | --- | --- | --- | --- |
| LY27 | 1 | 15-Jul | 31.22 | 75.98 | 21.21±0.13 b | 39.60±3.12 ab | 17.65±0.35 a |
| LY27 | 2 | 22-Jul | 31.10 | 76.31 | 22.36±0.66 b | 30.97±5.77 b | 20.32±3.16 a |
| LY27 | 3 | 7-Jul | 29.13 | 82.23 | 24.61±0.01 a | 49.20±6.18 a | 7.70±2.36 b |
| LY6 | 1 | 8-Jul | 29.43 | 80.68 | 23.98±0.52 a | 58.27±4.07 a | 8.82±0.93 b |
| LY6 | 2 | 13-Jul | 31.17 | 76.37 | 22.77±0.59 b | 53.02±2.13 a | 21.38±8.76 a |
| LY6 | 3 | 18-Jul | 30.96 | 75.79 | 24.29±0.24 a | 56.68±2.44 a | 8.90±1.87 b |
| ZLY47 | 1 | 5-Jul | 28.90 | 83.63 | 25.38±0.46 a | 59.15±2.42 a | 15.56±4.36 b |
| ZLY47 | 2 | 18-Jul | 30.96 | 75.79 | 24.20±0.29 b | 52.08±1.81 b | 30.25±2.90 a |
| R168 | 1 | 20-Jun | 27.23 | 84.10 | 23.93±0.37 a | 64.83±0.16 a | 4.73±1.41 b |
| R168 | 2 | 30-Jun | 28.29 | 84.08 | 23.53±0.21 a | 64.03±1.07 a | 5.08±0.94 b |
| R168 | 3 | 11-Jul | 30.61 | 76.98 | 23.80±0.01 a | 64.16±0.94 a | 9.52±3.29 a |
| IR64 | 1 | 30-Jul | 29.08 | 83.27 | 25.37±1.43 a | 63.91±0.70 a | 10.25±2.07 b |
| IR64 | 2 | 12-Aug | 28.59 | 85.31 | 24.74±0.29 a | 61.38±1.30 a | 9.13±1.99 b |
| IR64 | 3 | 5-Aug | 28.69 | 84.95 | 25.35±1.27 a | 62.02±0.02 a | 14.73±1.75 a |
| 16343 | 1 | 1-Aug | 29.08 | 83.78 | 20.94±0.30 a | 59.70±2.89 ab | 18.42±8.24 c |
| 16343 | 2 | 7-Aug | 28.46 | 85.81 | 21.24±0.38 a | 63.74±1.06 a | 8.27±2.85 d |
| 16343 | 3 | 23-Jul | 31.02 | 76.97 | 19.52±0.38 b | 55.37±4.24 b | 35.63±2.61 a |
