# Supplementary material for: Detrimental effects of heat stress on grain weight and quality in rice (Oryza sativa L.) are aggravated by decreased relative humidity
Source: PeerJ. 2021 Apr 9;9:e11218. doi: 10.7717/peerj.11218 (PMC8040870; doi:10.7717/peerj.11218)
Supplement: Supplemental Information 2 [file peerj-09-11218-s002.doc]

Table S2 Heading date, average daily temperature during 15 days after heading(T15) and average daily relative humidity during 15 days after heading (RH15) of each cultivar

| Cultivars | Heading Date(d-m) | T15(°C) | RH15(%) |
| --- | --- | --- | --- |
| LY27 | 27-Jun | 27.51 | 87.20 |
| LY6H | 26-Jun | 27.38 | 89.33 |
| ZLY47 | 26-Jun | 27.38 | 89.33 |
| R168 | 30-Jun | 28.20 | 84.16 |
| 16343 | 5-Jul | 28.87 | 83.80 |
| IR64 | 7-Jul | 28.93 | 83.29 |
